# Patients with acute myocarditis and preserved systolic left ventricular function: comparison of global and regional longitudinal strain imaging by echocardiography with quantification of late gadolinium enhancement by CMR

**DOI:** 10.1007/s00392-021-01885-0

**Published:** 2021-06-04

**Authors:** Christine Meindl, Michael Paulus, Florian Poschenrieder, Florian Zeman, Lars S. Maier, Kurt Debl

**Affiliations:** 1 Department of Internal Medicine II, University Hospital Regensburg, 93053 Regensburg, Germany; 2 Institute of Radiology, University Hospital Regensburg, Regensburg, Germany; 3 Center for Clinical Studies, University Hospital Regensburg, Regensburg, Germany

**Keywords:** Myocarditis, CMR, Speckle tracking, Regional longitudinal strain

## Abstract

**Background:**

Conventional transthoracic echocardiography (TTE) does often not accurately reveal pathologies in patients with acute myocarditis and preserved left ventricular ejection fraction (LVEEF). Therefore, we investigated the diagnostic value of two-dimensional (2D) speckle tracking echocardiography compared to late gadolinium enhancement (LGE) by cardiac magnetic resonance (CMR) imaging in patients with acute myocarditis and normal global LVEF.

**Methods and results:**

31 patients (group 1) with the diagnosis of acute myocarditis confirmed by CMR according to the Lake Louise criteria and 20 healthy controls (group 2) were analyzed including global longitudinal strain (GLS) and regional longitudinal strain (RLS) derived by the bull’s eye plot. Although preserved LVEF was present in both groups, GLS was significantly lower in patients with acute myocarditis (group 1: GLS − 19.1 ± 1.8% vs. group 2: GLS − 22.1 ± 1.7%, *p* < 0.001). Compared to controls, lower RLS values were detected predominantly in the lateral, inferolateral, and inferior segments in patients with acute myocarditis. Additionally RLS values were significantly lower in segments without LGE.

**Conclusion:**

In patients with acute myocarditis and preserved LVEF, a significant reduction of GLS compared to healthy subjects was detected. Further RLS adds important information to the localization and extent of myocardial injury.

**Graphic abstract:**

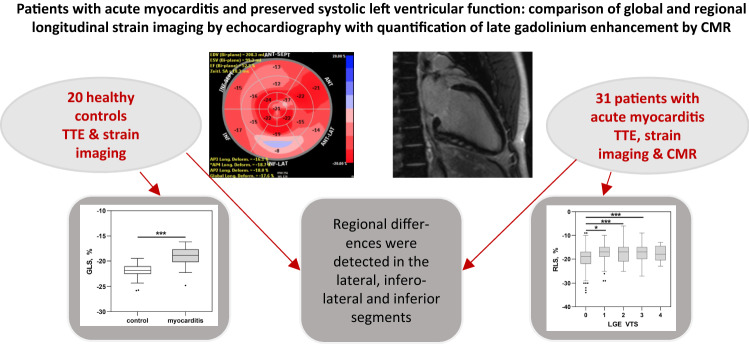

## Introduction

The clinical presentation of acute myocarditis is heterogeneous making the diagnosis challenging—especially in patients with preserved ejection fraction (LVEF) [1–5]. The course of acute myocarditis ranges from asymptomatic patients to cases with pseudo acute coronary syndrome, acute heart failure, ventricular arrhythmia, and sudden cardiac death [2].

The prognosis of acute myocarditis is also variable [6]. Acute myocarditis has been diagnosed in up to 12% of cases of sudden cardiac death [7] and the development of secondary dilated cardiomyopathy has been revealed in up to 30% of patients in long-term follow-up studies [8]. Concerning the outcome of patients with mild cases of acute myocarditis with preserved LVEF only limited data exist but scarce evidence support a benign course of this condition [9]. The ITAMY trial revealed different patterns of LGE by CMR in patients with preserved LVEF. The presence of LGE in the midwall layer of the anteroseptal segments was associated with a worse prognosis compared to other LGE patterns [10]. Gräni et al. also demonstrated that patients with midwall and septal LGE involvement had a higher risk for major adverse cardiac events (MACE). Additionally, a patchy distribution and LGE extent (per 10% increase) were detected as predictors of a higher risk of MACE [11].

While historically endomyocardial biopsy has represented the diagnostic gold standard of acute myocarditis [12], the current reference standard for noninvasive diagnosis of myocarditis is CMR imaging according to the Lake Louise consensus criteria and their updated version of 2018 [3, 8, 13].

LGE detected by CMR reflects myocardial injury, i.e., necrosis and fibrosis [3, 12]. Gräni et al. recently demonstrated an incremental prognostic value of LGE in risk stratifying patients with suspected myocarditis [11]. So far several studies have revealed a high specificity of LGE for the detection of myocardial injury in myocarditis [6, 14–17].

However, the quantification of LGE in patients with suspected myocarditis remains challenging because of the heterogeneity in presence, localization and intensity of LGE extent [8, 18]. In addition the accessibility of CMR is often limited and interpreting the diagnostic findings requires substantial experience. That is why the diagnosis of acute myocarditis by objective measurements remains challenging [19, 20].

Conventional TTE does often not accurately reveal pathologies in patients with acute myocarditis and preserved left ventricular ejection fraction (LVEF) [21]. Novel imaging modalities such as speckle tracking echocardiography enable the precise detection of subtle global and regional left ventricular dysfunction. Concerning strain imaging some methodological aspects warrant consideration. As a semiautomated method longitudinal strain analysis is accompanied by a learning curve and represents a potential source of measurement variability [22]. Further there still exist vendor-specific differences with respect to calculation of GLS values [23]. The selection of fiducial landmarks and segmental contouring is also essential to optimize strain imaging [22].

We investigated the diagnostic value of global longitudinal strain imaging by echocardiography compared to myocardial injury detected by CMR imaging in patients with acute myocarditis and normal global LVEF. Myocardial injury was diagnosed by the presence of LGE and myocardial edema in CMR. To quantify myocardial injury established visual presence scores were used.

## Methods

### Study population

We retrospectively identified 31 patients with acute myocarditis and preserved LVEF admitted to the University Hospital Regensburg between November 2015 and November 2019. The diagnosis of myocarditis was confirmed by typical clinical presentation and by typical findings in CMR. The so-called typical clinical presentation comprises “infarct-like” myocarditis including chest pain, elevation of troponin and creatinkinase levels, ECG abnormalities, and preceding signs of infection.

In all the patients included typical symptoms as chest pain and elevation of troponin and creatinkinase (CK) levels were present. In 74.2% of patients ECG abnormalities were found and 83.9% had preceding signs of infection. CMR and TTE were conducted in the acute phase of myocarditis. Patients with a history of severe symptoms, e.g., sudden cardiac death, heart failure or reduced LVEF were excluded. In addition patients aged < 18 years and patients with poor TTE image quality were not included. Clinical data and blood samples of all participants were analyzed. The study was approved by the local ethics committee.

20 healthy age- and gender-matched subjects were recruited to serve as a control group. The control group did not have a history of myocarditis and other cardiac diseases. All healthy volunteers gave written informed consent and were investigated by complete TTE. No external funding was obtained to support the study.

### Echocardiography

TTE was performed in the first few days of acute myocarditis using IE33and Epiq CVx (Philips Medical Systems, Amsterdam, The Netherlands) ultrasound systems as well as S5-1 or X5-1 (Philips Medical Systems, Amsterdam, The Netherlands) transducers. Routine two-dimensional (2D) cine loops were obtained and three apical views (four-chamber, two-chamber, and three-chamber view) were stored digitally. LVEF was calculated by a biplane Simpson’s method from apical four- and two-chamber views or if not applicable from an apical four-chamber view. TTEs were analyzed offline using the IntelliSpace QLAB software (Philips Medical Systems, Amsterdam, The Netherlands) by two experienced observers (C.M., K.D.). The TTEs were studied independently by each observer.

### Speckle tracking analysis

Speckle tracking analysis was performed on a frame-by-frame basis by automatic tracking of acoustic markers (speckles) throughout the cardiac cycle. The duration of systole was defined in the apical three-chamber view by marking the aortic valve opening and closure. The myocardial borders were traced from the apical four-, two-, and three-chamber views in the end-systolic frame of the 2D images to analyze global longitudinal strain (GLS).

In accordance with the consensus document of the EACVI/ASE/Industry the region of interest (ROI) was defined by the endocardial border which symbolizes the inner contour of the myocardium [24]. If necessary, endocardial borderlines were manually adapted and motion tracking was performed automatically by the IntelliSpace QLAB software.

**Fig. 1:**
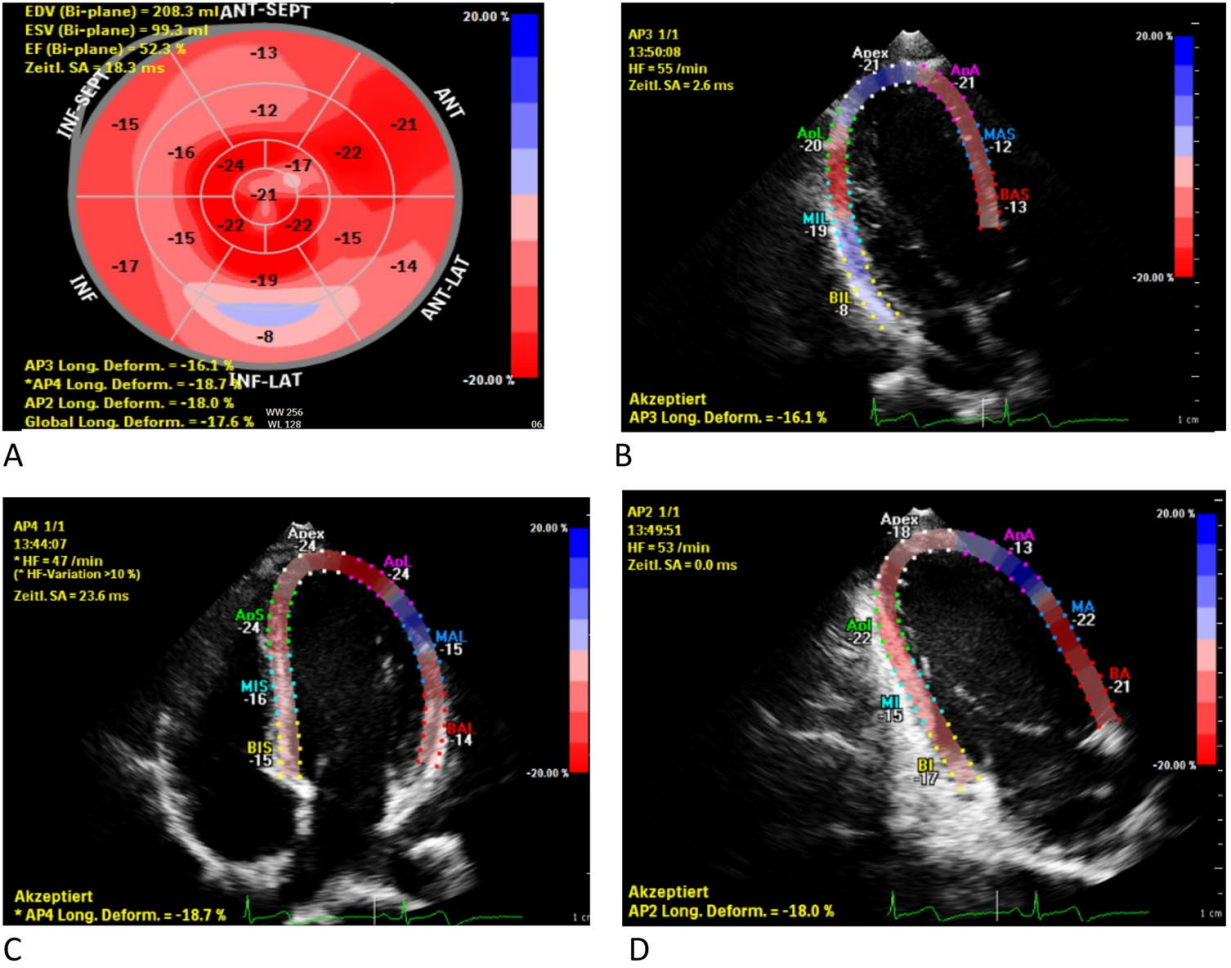
**a** Example of a bull’s eye plot with reduced GLS (− 17.6%) and segmental abnormalities pronounced in the inferior, inferolateral, and anterolateral segments. **b**–**d** Corresponding apical 3 (**b**), 4 (**c**) and 2 (**d**) chamber view with reduced longitudinal strain values

Peak systolic strain was determined as the maximum value of the peak negative strain during systole. By assessing the peak systolic longitudinal strain in all 17 longitudinal segments global longitudinal strain was provided by the software as the average value of the different segments (Fig. 1b–d). To depict regional differences according to the commonly used 17-segment model [24] the software presented the results in the form of bull’s eye plot (Fig. 1a).

### Cardiac magnetic resonance imaging

**Fig. 2:**
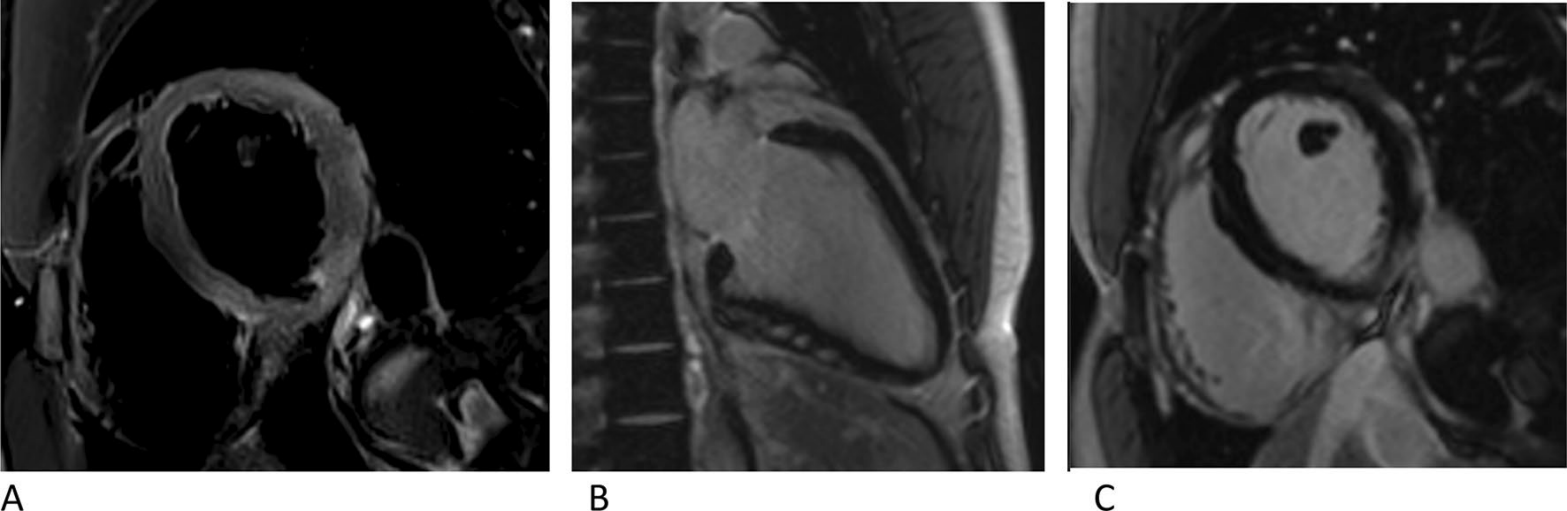
A T2STIR CMR image of the same patient as in Fig. 1**a**–**d** with edema in the inferior and inferolateral segments. **b**, **c** Example of a corresponding CMR long axis (**b**) and short axis (**c**) view with LGE in the inferior and inferolateral segments

CMR was performed in the first few days of acute myocarditis on a 1.5 or 3 Tesla scanner (Magnetom Avanto or Magnetom Skyra, Siemens Healthcare, Erlangen, Germany) using a 32-channel phased-array receiver coil. CMR images were acquired during breath hold and with ECG-gating. The CMR imaging protocol included short axis fat-saturated T2-weighted sequences searching for myocardial edema and postcontrast late gadolinium-enhanced T1-weighted phase-sensitive inversion recovery (PSIR) sequences in short axis, vertical long axis, and horizontal long axis view for the detection of myocardial injury (LGE). T2STIR imaging was performed in all patients and analyzed visually, but not further quantified. According to the updated Lake Louise criteria we used T2-weighted images to search for edema and quantified LGE to detect non-ischemic myocardial injury [13]. Gadovist (0.2 mmol/kg body weight, Bayer Healthcare, Leverkusen, Germany) was used as a contrast agent. Figure 2a–c shows an example of acute myocarditis in CMR.

As already described by Gräni et al. LGE extent was determined visually by the 17-segment model using two different scores [18]: The visual presence score (LGE-VPS) comprises LGE being present or not in a segment (maximum score 17) and the visual transmurality score (LGE-VTS) summing up the transmural extent of LGE per segment, assessed by a five-point scale (0 = no LGE, 1 ≤ 25% transmurality, 2 = 26–50% transmurality, 3 = 51–75% transmurality, 4 = 76–100% transmurality, maximum score 68) [18].

### Statistical analysis

Statistical analysis was performed using SPSS Version 25 (International Business Machines Corporation, IBM, Armonk, NY USA). Categorical data are presented as absolute and relative frequencies whereas continuous variables are presented as mean ± standard deviation or as median (interquartile range) depending on the underlying distribution. Differences between continuous variables in paired data were tested with a paired *t* test, continuous variables in unpaired data were compared with an unpaired *t* test. Further the chi-squared test was used for the gender comparison between the groups. A *p* value of < 0.05 was considered statistically significant.

## Results

Baseline characteristics of the study population are depicted in Table 1. Mean age of patients with acute myocarditis was 29 ± 9 years compared to 33 ± 4 years in the control group (*p* = 0.06). No significant differences were revealed concerning gender or comorbidities like arterial hypertension or valvular heart disease. BMI levels were significantly lower in the control group than in patients with myocarditis (BMI 23.7 ± 2.4 kg/m^2^ vs. 25.8 ± 3.6 kg/m^2^, *p* = 0.025). 83.9% of patients with acute myocarditis had preceding signs of infection and in 74.2% ECG abnormalities were present. Coronary angiography was performed in 51.6% of patients with myocarditis and obstructive coronary artery disease was excluded in all patients. CMR has shown to provide a high sensitivity and specificity in cases of “infarct-like” myocarditis diagnosed according to the Lake Louise criteria. In our tertiary care center we are able to offer immediate access to CMR. Thus we could avoid coronary angiography in young patients without atherosclerotic risk factors [13].

**Table 1:** Baseline characteristics of the study population

	Myocarditis (*n* = 31)	Control (*n* = 20)	*p* value
Age, years ± SD	29 ± 9	33 ± 4	0.060
Female gender, *n* (%)	3 (9.7)	1 (5.0)	1.000
BMI, kg/m^2^ ± SD	25.8 ± 3.6	23.7 ± 2.4	**0.025**
Heart rate, bpm ± SD	70 ± 13	71 ± 13	0.813
Arterial hypertension, *n* (%)	1 (3.2)	0	1.000
Diabetes mellitus, *n*	0	0	
Troponin (pg/ml)	8310 (1490–26,800)	n.a	
CRP (mg/l)	36.4 (10.2–83.4)	n.a	
ECG abnormalities, *n* (%)	23 (74.2)	n.a	
Preceding signs of infection, *n* (%)	26 (83.9)	n.a	
Coronary angiography performed, *n* (%)	16 (51.6)	n.a	
Diagnosis of obstructive coronary artery disease, *n*	0	n.a	

*SD* standard deviation, *BMI* body mass index, *CRP* C-reactive protein, *ECG* electrocardiogram

Usually several measurements of troponin and CRP have been performed in our institution. Peak troponin and creatinkinase levels were selected to present the maximum extent of myocardial injury. Median peak troponin level was 8310 pg/ml (min–max 1490–26,800) and median maximum C-reactive protein (CRP) was 36.4 mg/l (10.2–83.4) in patients with acute myocarditis.

Ejection fraction was preserved in the myocarditis group (LVEF 58 ± 4%) as well as in the control group (LVEF 60 ± 4%, *p* = 0.135, Table 2). Diastolic posterior wall thickness measured by echocardiography was significantly higher in patients with myocarditis (10 ± 1 mm) than in the control group (9 ± 1 mm, *p* = 0.021). Further diastolic dysfunction measured by E/E’ mean was significantly deteriorated in the myocarditis group compared to the control group (Table 2).

**Table 2:** Echocardiographic results

	Myocarditis (*n* = 31)	Control (*n* = 20)	*p* value
LVEF biplane, % ± SD	58 ± 4	60 ± 4	0.135
IVSd, mm ± SD	10 ± 2	9 ± 2	0.117
PWd, mm ± SD	10 ± 1	9 ± 1	**0.021**
LVESD, mm ± SD	35 ± 6	34 ± 4	0.784
LVEDD, mm ± SD	49 ± 6	48 ± 4	0.595
LV mass, g ± SD	154 ± 46	131 ± 39	0.082
LV end-diastolic volume, ml	113 ± 31	116 ± 23	0.681
LV end-systolic volume, ml	49 ± 16	48 ± 12	0.956
E/E’ mean	6.5 ± 1.7	5.5 ± 1.0	**0.024**
RVEDD, mm ± SD	33 ± 5	34 ± 2	0.158
Global longitudinal strain, % ± SD	− 19.1 ± 1.8	− 22.1 ± 1.7	** < 0.001**

Continuous variables with normal distribution are expressed as mean ± SD

*LVEF* left ventricular ejection fraction, *SD* standard deviation, *IVSd* interventricular septum diastolic thickness, *PWd* posterior wall diastolic thickness, *LVESD* left ventricular end-systolic diameter, *LVEDD* left ventricular end-diastolic diameter, *LV* left ventricle, *RVEDD* right ventricular end-diastolic diameter

**Fig. 3:**
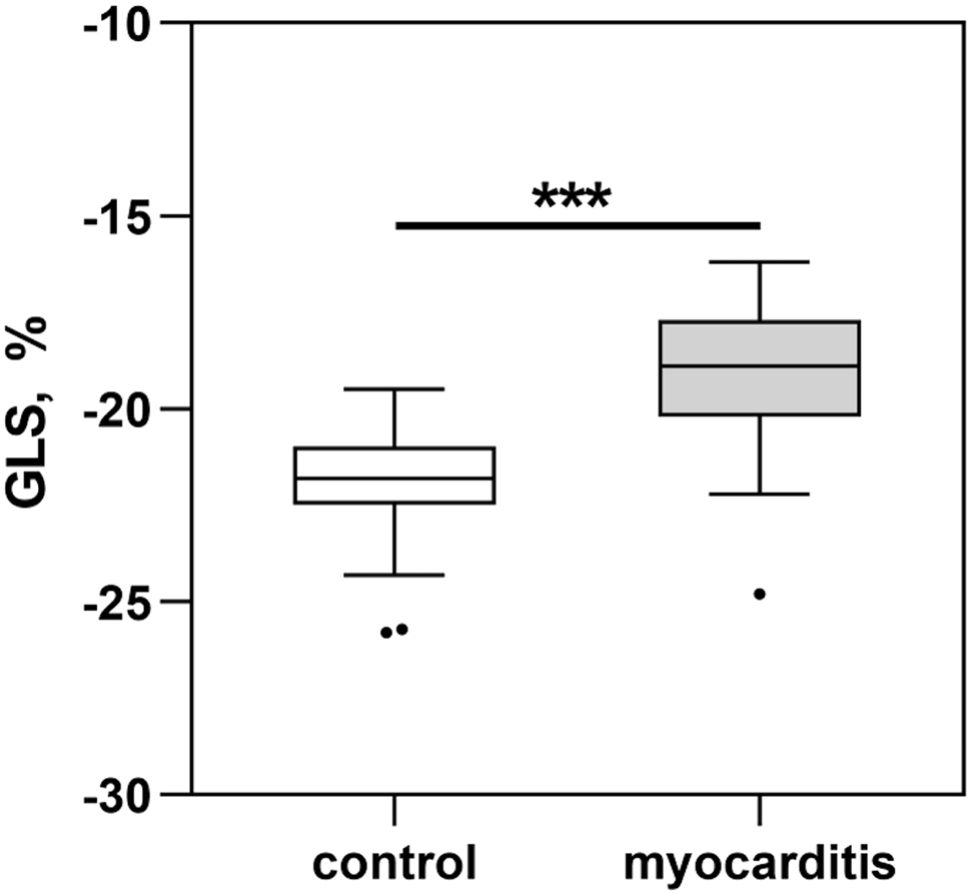
Global longitudinal strain in the control and myocarditis group. Data are shown as Tukey boxplots. ***p < 0.001

Despite preserved ejection fraction in both groups GLS was significantly decreased in patients with acute myocarditis (GLS − 19.1 ± 1.8%) compared to the control group (GLS − 22.1 ± 1.7%, *p* < 0.001, Fig. 3).

**Fig. 4:**
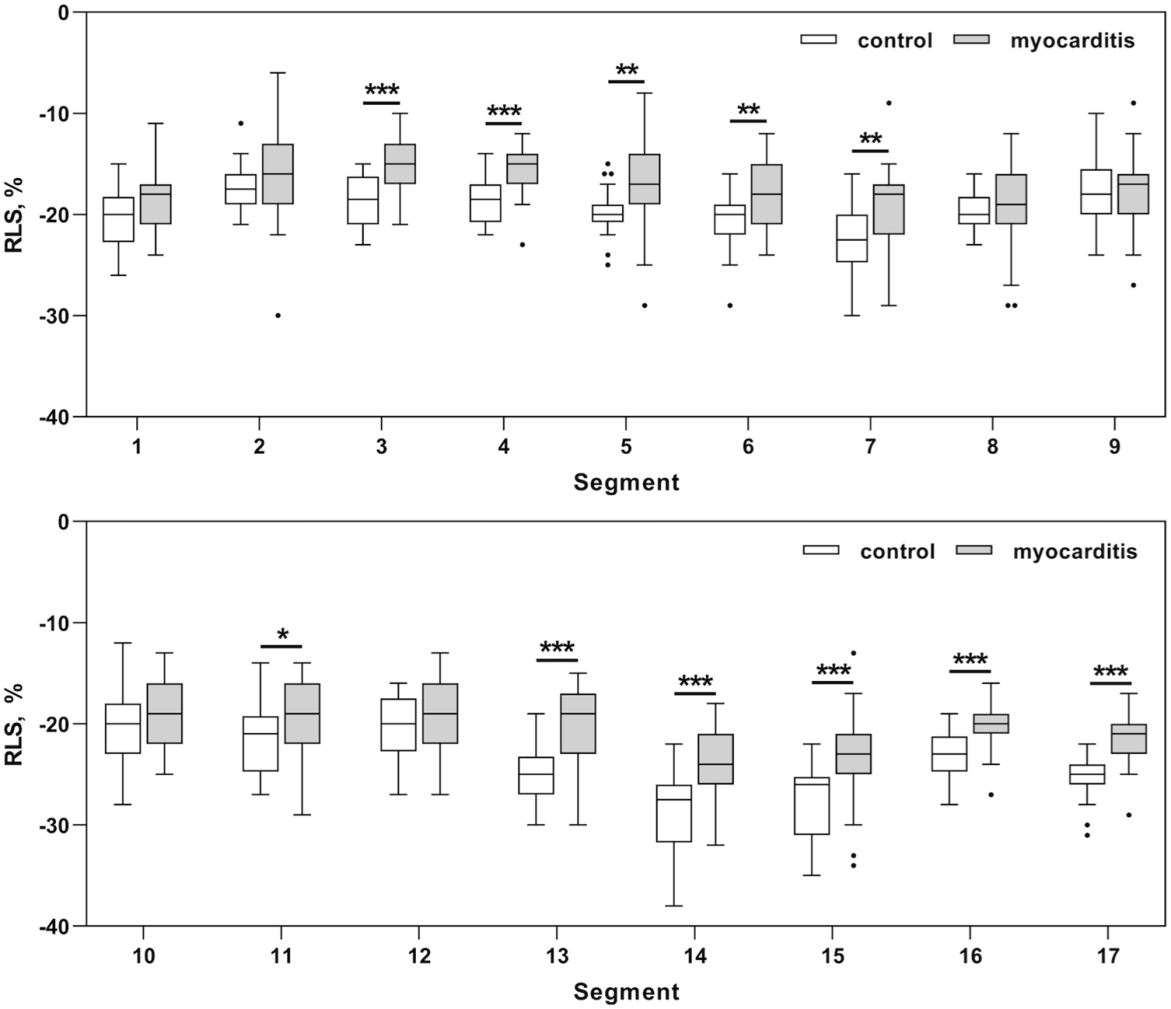
Regional longitudinal strain of individual segments in the control and myocarditis group. Data are shown as Tukey boxplots.*p < 0.05 **p < 0.01 ***p < 0.001

Figure 4 shows the values of segmental longitudinal strain according to the AHA 17-segment model. As depicted in Fig. 4 regional strain values of the myocarditis group were significantly decreased in several segments compared to the control group predominantly in the lateral, inferolateral, and inferior segments.

**Fig. 5:**
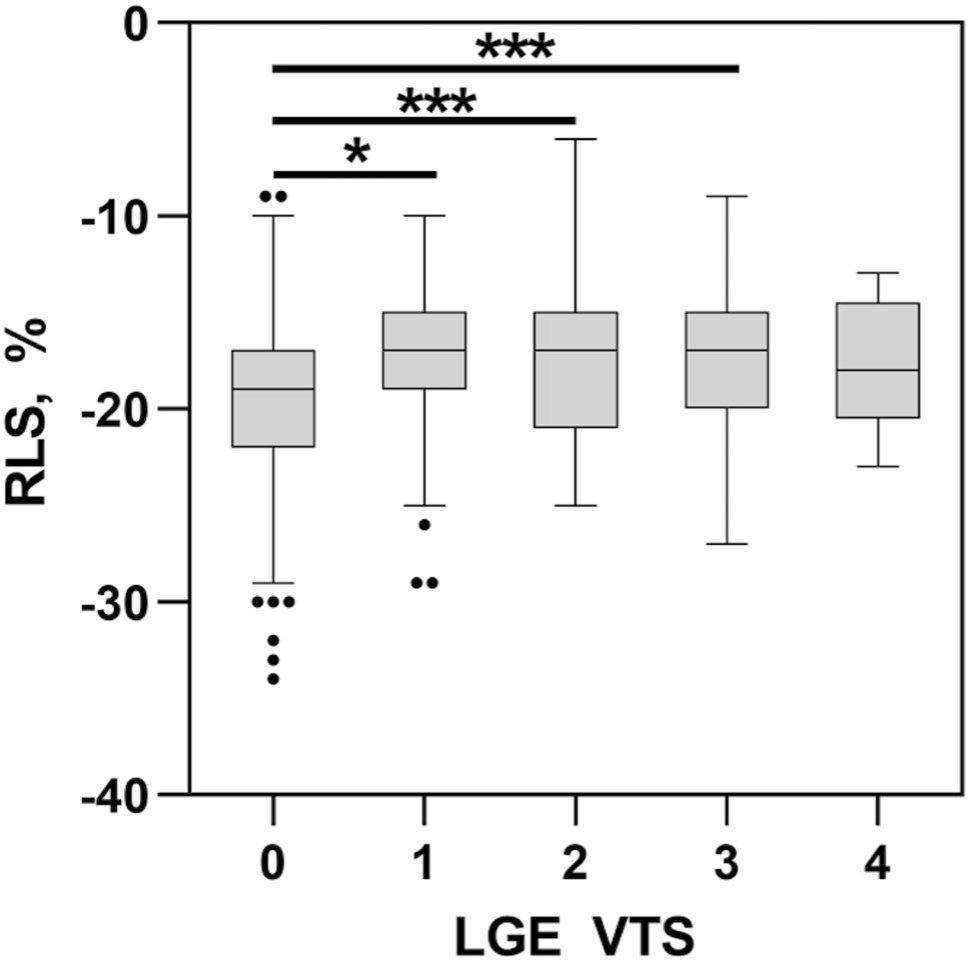
Regional longitudinal strain stratified by LGE-VTS score in cardiac MRI. Data are shown as Tukey boxplots. *p < 0.05 ***p < 0.001

With regard to the CMR results (Table 3) of the myocarditis group mean LGE-VPS was 6.1 ± 2.9 (maximum score 17) and mean LGE-VTS was 13.6 ± 7.3 (maximum score 68). Focusing on the regional presence, LGE was most frequently detected in the inferior and inferolateral segments (Table 3). LGE extent ≤ 25–75% was associated was impaired RLS (Fig. 5). In contrast LGE with extent of 76–100% did not show significant results.

**Table 3:** CMR data of the patients with myocarditis (*n* = 31)

LVEF, % ± SD	58 ± 7
LGE visual presence score ± SD	6.1 ± 2.9
LGE visual transmurality score ± SD	13.6 ± 7.3
Regional LGE presence	*n* (%)
Segment 1	5 (16.1)
Segment 2	4 (12.9)
Segment 3	20 (64.5)
**Segment 4**	**26 (83.9)**
**Segment 5**	**30 (96.8)**
Segment 6	12 (38.7)
Segment 7	7 (22.6)
Segment 8	5 (16.1)
Segment 9	7 (22.6)
Segment 10	6 (19.4)
Segment 11	27 (87.1)
Segment 12	14 (45.2)
Segment 13	3 (9.7)
Segment 14	4 (12.9)
Segment 15	4 (12.9)
Segment 16	13 (41.9)
Segment 17	3 (9.7)

## Discussion

In the present study, we focused on patients with acute myocarditis and preserved ejection fraction. The major findings of this study are:

- Despite normal LVEF values GLS was significantly reduced in the myocarditis group compared to a healthy control group suggesting subtle pathologies.
- In patients with acute myocarditis lower RLS values compared to healthy subjects were detected predominantly in the lateral, inferolateral, and inferior segments
- Further significant associations between RLS measured by echocardiography and semi-quantitative analysis of myocardial injury by CMR (LGE-VTS score) were revealed.

Gräni et al. demonstrated an incremental prognostic benefit of LGE in risk stratifying patients with suspected myocarditis [11]. Despite this fact an optimal method of LGE quantification in patients with suspected myocarditis is currently not available. Better characterization of LGE patterns is necessary as the presence, localization and intensity of LGE varies in patients with myocarditis [8].

Therefore, Gräni et al. intended to compare LGE quantification methods including thresholding by 2, 3, 4, 5, 6 or 7 standard deviations above remote myocardium, the full width at half maximum (FWHM) technique, and visual quantification in patients with suspected myocarditis [11]. Gräni et al. demonstrated that FWHM had the highest technical consistency and the strongest prognostic correlation with major adverse cardiovascular events (MACE) of all LGE quantification methods in patients with suspected myocarditis [18]. However, visual qualitative assessment of LGE extent using the LGE-VPS score turned out to be a reliable alternative prognosticating method showing an excellent intra- and inter-rater variability [18].

Therefore, we chose the LGE-VTS and LGE-VPS score to quantify myocardial injury in patients with acute myocarditis. In the present study, we could demonstrate significant associations between regional longitudinal strain and LGE-VTS score. The reason why LGE extent of 76–100% did not show significant results is probably the low number of segments with LGE extent of 76–100% (17 of 527 segments).

In another study by Mewton et al. including 41 patients LGE extent—quantified by visual transmurality score—represented an independent predictor of MACE with Hazard Ratio (HR) 1.42 [25]. Additionally, Barone-Rochette et al. detected a trend towards worse outcome in patients with suspected myocarditis with higher LGE extent scored by a simplified quantitative score (SQS), similar to the LGE-VTS score described by Gräni et al. [26].

Based on this evidence, it is important not only to describe the presence but also the extent of LGE in patients with acute myocarditis for instance using visual LGE quantification scores.

In addition to myocardial fibrosis edema represents another important diagnostic criterion in acute myocarditis. Løgstrup et al. showed that in patients with acute myocarditis edema was mainly localized in the infero-postero-lateral segments assessed by CMR. In accordance with the CMR results segmental strain was also predominantly reduced in the infero-postero-lateral segments in 2D echocardiography [20]. The present study confirms regional differences with inferior and inferolateral segments being the most affected by myocardial injury in CMR and reduced segmental longitudinal strain in 2D echocardiography. Our study also revealed that diastolic posterior wall thickness measured by echocardiography was significantly higher in patients with myocarditis than in the control group potentially indicating myocardial edema in the myocarditis group. Further diastolic dysfunction was significantly deteriorated in the myocarditis group compared to the control group which may also be caused by structural changes in the myocarditis group.

Our speckle tracking results also showed significant differences in the apical segments between the myocarditis and the control group. However, the strain values in the apical segments were in the normal range in both groups.

As described by Løgstrup et al. our results suggest global and segmental strain imaging adding valuable information compared to conventional 2D echocardiography in patients with acute myocarditis [20]. Escher et al. could show that patients with acute myocarditis (*n* = 25) diagnosed by endomyocardial biopsy had a reduction in global longitudinal strain during the acute course of myocarditis. At follow-up during a period of 6.2 months in eight patients inflammation persisted and global longitudinal strain and strain rate were significantly lower in these patients compared to patients without inflammation [27].

Therefore, deformation analysis by speckle tracking echocardiography should be added to routine diagnostics in patients with suspected acute myocarditis. A reduction of global longitudinal strain in addition with abnormalities in the lateral, inferolateral and inferior segments in the bull’s eye plot should be added to an algorithm for the diagnosis of myocarditis. The importance of speckle tracking echocardiography is even more pronounced in institutions with limited access to CMR.

Caspar et al. detected myocardial dysfunction by 2D and 3D speckle tracking analysis in patients late after an acute episode of myocarditis and preserved LV function. Mean delay between the diagnosis of acute myocarditis and follow-up echocardiography was 21.7 ± 23.4 months [3]. However, these results demonstrate that strain imaging represents a valuable tool to reveal subtle pathologies during follow-up visits in patients with a history of myocarditis.

The present study included patients with acute myocarditis and speckle tracking analysis as well as CMR were performed in the first few days of myocarditis. Whether these pathologies in strain imaging and CMR remain during follow-up visits should be further investigated.

Concerning strain imaging some methodological aspects warrant consideration. As a semiautomated method longitudinal strain analysis is accompanied by a learning curve and represents a potential source of measurement variability [22]. Further there still exist vendor-specific differences with respect to calculation of GLS values [23]. The selection of fiducial landmarks and segmental contouring is also essential to optimize strain imaging [22].

Further new evolving techniques like feature tracking by CMR could add valuable information in patients with suspected myocarditis. Recently Gräni et al. demonstrated an independent and incremental prognostic value over clinical features, LVEF and LGE in patients with myocarditis by the use of CMR feature tracking [28].

Some limitations of the present study warrant consideration:

First, our study represents a single-center experience with a retrospective study design. Second, we did not analyze circumferential and radial strain parameters. We chose to investigate the longitudinal strain parameters as it is known that in patients with acute myocarditis the most affected myocardial layer is the subepicardium and there the fibers are mainly directed in a longitudinal way [3, 29]. Further global longitudinal strain is the most robust parameter concerning speckle tracking. The study might also have revealed additional information if we had used circumferential or radial strain to find out more about the myocardial fiber architecture.

Third, the 1.5 and 3.0 Tesla CMR systems were used to evaluate patients with suspected myocarditis which may possibly influence LGE quantification. Fourth, because of the retrospective study design we were not able to perform T2-mapping as a novel technique for quantifying myocardial edema.

Fifth, the members of the control group had significantly lower BMI values compared to the patients with myocarditis albeit none of the groups had the diagnosis of obesity.

## Conclusion

In patients with acute myocarditis and preserved LVEF a significant reduction of GLS compared to healthy subjects was detected. Furthermore RLS adds important information to conventional TTE which could otherwise only be derived by CMR.

## Data Availability

The data underlying this article will be shared on reasonable request to the corresponding author.

## References

[1] Gore I, Saphir O (1947). Myocarditis; a classification of 1402 cases. Am Heart J.

[2] Caforio AL (2015). Clinical presentation and diagnosis of myocarditis. Heart.

[3] Caspar T (2017). Late detection of left ventricular dysfunction using two-dimensional and three-dimensional speckle-tracking echocardiography in patients with history of nonsevere acute myocarditis. J Am Soc Echocardiogr.

[4] Camastra GS (2007). Late enhancement detected by cardiac magnetic resonance imaging in acute myocarditis mimicking acute myocardial infarction: location patterns and lack of correlation with systolic function. J Cardiovasc Med (Hagerstown).

[5] Khoo NS (2012). Altered left ventricular tissue velocities, deformation and twist in children and young adults with acute myocarditis and normal ejection fraction. J Am Soc Echocardiogr.

[6] Mahrholdt H (2006). Presentation, patterns of myocardial damage, and clinical course of viral myocarditis. Circulation.

[7] Basso C (2001). Postmortem diagnosis in sudden cardiac death victims: macroscopic, microscopic and molecular findings. Cardiovasc Res.

[8] Friedrich MG (2009). Cardiovascular magnetic resonance in myocarditis: a JACC white paper. J Am Coll Cardiol.

[9] Imazio M (2013). Good prognosis for pericarditis with and without myocardial involvement: results from a multicenter, prospective cohort study. Circulation.

[10] Aquaro GD (2017). Cardiac MR with late gadolinium enhancement in acute myocarditis with preserved systolic function: ITAMY study. J Am Coll Cardiol.

[11] Grani C (2017). Prognostic value of cardiac magnetic resonance tissue characterization in risk stratifying patients with suspected myocarditis. J Am Coll Cardiol.

[12] Caforio AL et al (2013) Current state of knowledge on aetiology, diagnosis, management, and therapy of myocarditis: a position statement of the European Society of Cardiology Working Group on Myocardial and Pericardial Diseases. Eur Heart J 34(33):2636–48, 2648a–2648d 10.1093/eurheartj/eht210 23824828

[13] Ferreira VM (2018). Cardiovascular magnetic resonance in nonischemic myocardial inflammation: expert recommendations. J Am Coll Cardiol.

[14] Mahrholdt H (2004). Cardiovascular magnetic resonance assessment of human myocarditis: a comparison to histology and molecular pathology. Circulation.

[15] Abdel-Aty H (2005). Diagnostic performance of cardiovascular magnetic resonance in patients with suspected acute myocarditis: comparison of different approaches. J Am Coll Cardiol.

[16] Laissy JP (2005). Differentiating acute myocardial infarction from myocarditis: diagnostic value of early- and delayed-perfusion cardiac MR imaging. Radiology.

[17] Ingkanisorn WP (2006). Cardiac magnetic resonance appearance of myocarditis caused by high dose IL-2: similarities to community-acquired myocarditis. J Cardiovasc Magn Reson.

[18] Grani C (2019). Comparison of myocardial fibrosis quantification methods by cardiovascular magnetic resonance imaging for risk stratification of patients with suspected myocarditis. J Cardiovasc Magn Reson.

[19] Dennert R, Crijns HJ, Heymans S (2008). Acute viral myocarditis. Eur Heart J.

[20] Logstrup BB (2016). Myocardial oedema in acute myocarditis detected by echocardiographic 2D myocardial deformation analysis. Eur Heart J Cardiovasc Imaging.

[21] Goitein O (2009). Acute myocarditis: noninvasive evaluation with cardiac MRI and transthoracic echocardiography. AJR Am J Roentgenol.

[22] Collier P, Phelan D, Klein A (2017). A test in context: myocardial strain measured by speckle-tracking echocardiography. J Am Coll Cardiol.

[23] Lang RM (2015). Recommendations for cardiac chamber quantification by echocardiography in adults: an update from the American Society of Echocardiography and the European Association of Cardiovascular Imaging. J Am Soc Echocardiogr.

[24] Voigt JU (2015). Definitions for a common standard for 2D speckle tracking echocardiography: consensus document of the EACVI/ASE/Industry Task Force to standardize deformation imaging. Eur Heart J Cardiovasc Imaging.

[25] Mewton N (2015). Myocardial biomarkers and delayed enhanced cardiac magnetic resonance relationship in clinically suspected myocarditis and insight on clinical outcome. J Cardiovasc Med (Hagerstown).

[26] Barone-Rochette G (2014). Potentially simple score of late gadolinium enhancement cardiac MR in acute myocarditis outcome. J Magn Reson Imaging.

[27] Escher F (2013). New echocardiographic findings correlate with intramyocardial inflammation in endomyocardial biopsies of patients with acute myocarditis and inflammatory cardiomyopathy. Mediators Inflamm.

[28] Fischer K (2020). Feature tracking myocardial strain incrementally improves prognostication in myocarditis beyond traditional CMR imaging features. JACC Cardiovasc Imaging.

[29] Greenbaum RA (1981). Left ventricular fibre architecture in man. Br Heart J.

